# Sham-derived effects and the minimal reliability of theta burst stimulation

**DOI:** 10.1038/s41598-021-98751-w

**Published:** 2021-10-27

**Authors:** P. O. Boucher, R. A. Ozdemir, D. Momi, M. J. Burke, A. Jannati, P. J. Fried, A. Pascual-Leone, M. M. Shafi, Emiliano Santarnecchi

**Affiliations:** 1 Berenson-Allen Center for Noninvasive Brain Stimulation, Division of Interventional Cognitive Neurology, Beth Israel Deaconess Medical Center, Harvard Medical School, Boston, MA USA; 2 Department of Neurology, Harvard Medical School, Boston, MA USA; 3 Hinda and Arthur Marcus Institute for Aging Research and Deanna and Sidney Wolk Center for Memory Health, Hebrew SeniorLife, Boston, MA USA; 4 Guttmann Brain Health Institute, Universitat Autonoma, Barcelona, Spain; 5 Siena Brain Investigation & Neuromodulation Lab (Si-BIN Lab), Department of Medecine, Surgery and Neuroscience, Neurology and Clinical Neurophysiology Unit, Siena School of Medecine, Siena, Italy; 6 Harquail Centre for Neuromodulation and Hurvitz Brain Sciences Program, Sunnybrook Research Institute, Toronto, ON Canada; 7 Neuromodulation Program and Division of Epilepsy and Clinical Neurophysiology, Department of Neurology, Boston Children’s Hospital, Harvard Medical School, Boston, MA USA

**Keywords:** Neuromuscular junction, Excitability

## Abstract

Theta-burst stimulation (TBS) is a patterned form of repetitive transcranial magnetic stimulation (rTMS) that has been used to induce long-term modulation (*plasticity*) of corticospinal excitability in a drastically shorter duration protocol than conventional rTMS protocols. In this study we tested the reliability of the effects of two well defined TBS protocols, continuous TBS (cTBS) and intermittent TBS (iTBS), especially in relation to sham TBS, within and across the same 24 participants. All TBS protocols were repeated after approximately 1 month to assess the magnitude and reliability of the modulatory effects of each TBS protocol. Baseline and post-TBS changes in motor evoked potentials (MEP—measure of corticospinal excitability) amplitudes were compared across the cTBS, iTBS and sham TBS protocols and between the initial and retest visits. Overall, across participants, at the initial visit, iTBS facilitated MEPs as compared to baseline excitability, with sham eliciting the same effect. cTBS did not show a significant suppression of excitability compared to baseline MEPs at either visit, and even facilitated MEPs above baseline excitability at a single time point during the repeat visit. Otherwise, effects of TBS were generally diminished in the repeat visit, with iTBS and sham TBS replicating facilitation of MEPs above baseline excitability at similar time points. However, no protocol demonstrated consistent intra-individual modulation of corticospinal excitability upon retest. As the first study to test both iTBS and cTBS against sham TBS across repeat visits, our findings challenge the efficacy and reliability of TBS protocols and emphasize the importance of accounting for sham effects of TBS. Furthermore, given that therapeutic effects of TBS are hypothetically derived from consistent and repeated modulation of brain activity, the non-replicability of plasticity and sham effects call into question these basic mechanisms.

## Introduction

There is ample evidence to support the notion that suprathreshold and subthreshold repetitive transcranial magnetic stimulation (TMS) protocols modulate corticospinal excitability beyond the duration of the stimulation itself^1–4^. Neuromodulatory effects of trains of repetitive TMS (rTMS) can be evaluated by comparing the amplitude of TMS-induced motor evoked potentials (MEPs) before and after the stimulation trains^5^. The duration of the rTMS aftereffects depends on the intensity, frequency, and pattern of the TMS pulses and may be influenced by genetics^6^ and state-dependent effects^7,8^. Theta burst stimulation (TBS), is a particular type of rTMS, developed to be similar to protocols shown to induce long-term potentiation (LTP) or long-term depression (LTD) in animal models^3^. A particularly appealing feature of TBS is the reported long duration of neuromodulatory effects despite the brief duration of the protocols^9^.

Despite the potential of TBS protocols and their increasingly widespread use^9–11^, a number of recent studies have challenged the efficacy and reliability of their effects on corticomotor excitability^1,12^. Specifically, several recent studies have tested the reliability of TBS protocols within and between participants^2,13–18^ with varying results. Given that TBS protocols, especially iTBS, are increasingly used in therapeutic applications of TMS^9,10^, its reliability remains an important question^2^. The effectiveness and reliability of TBS protocols should be expressly tested against a robust sham TBS procedure in order to disentangle time-varying characteristics of MEPs from veridical TBS-induced modulation. Unfortunately, to date, only three such studies exist each differing in methodology, with one study where sham was conducted by holding the stimulating coil tangentially to the scalp^14^, another using a sham coil^13^, and the third using active vertex stimulation as a control^19^. Of concern is that two of these studies did not report using any kind of mask, method of replicating skin sensations caused by TBS, thus participants could be unblinded by being able to distinguish protocols^20^. The other used vertex stimulation as a control, a form of active masking, that has been observed to have widespread and complex effects on brain activity^21^. Raising its own concerns of possible indirect and poorly understood effects on cortical excitability.

**Figure 1:**
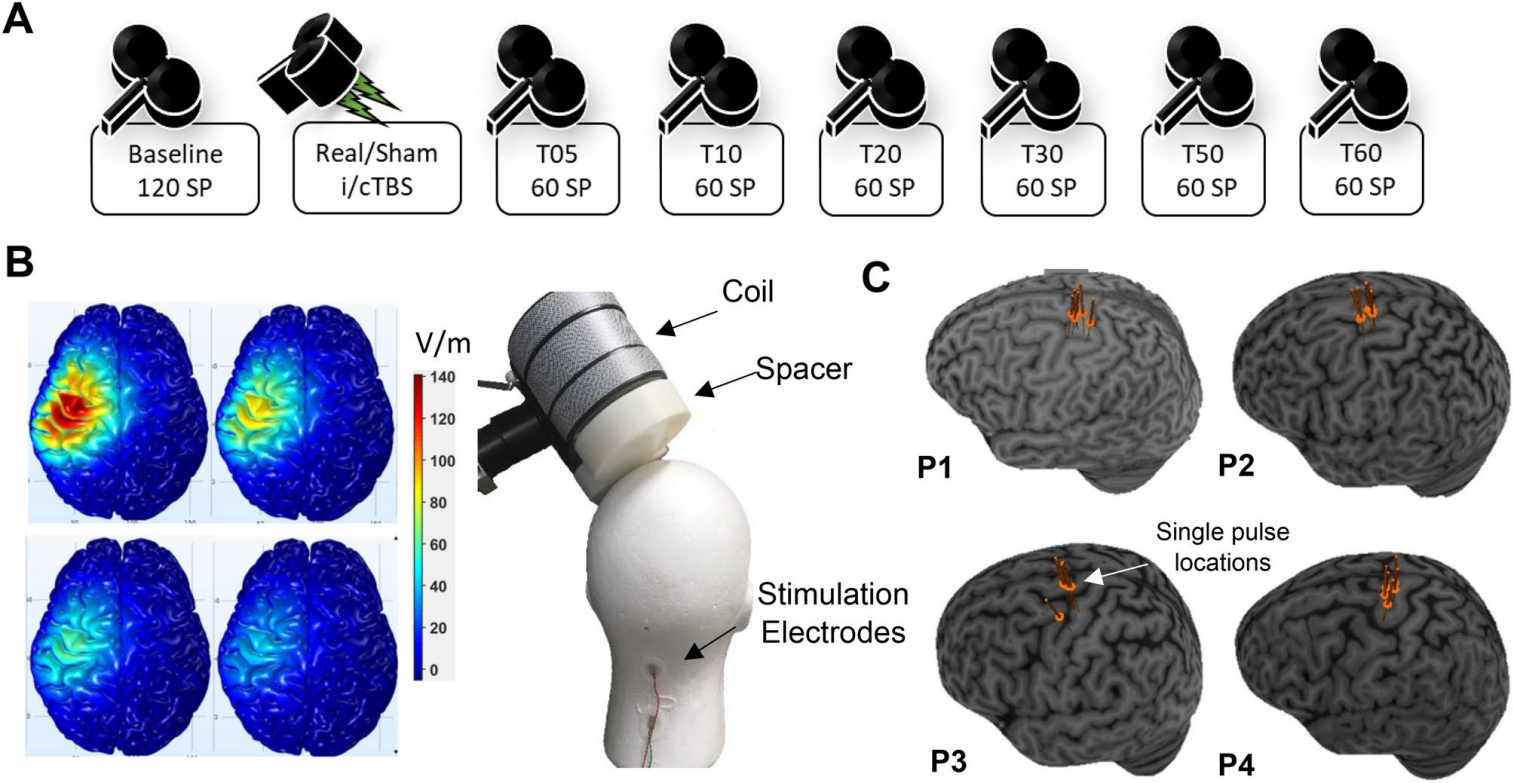
Experimental design. (**A**) Sequence of plasticity protocol in each visit (*SP* single pulses). (**B**) Left Panel: Magnitude of the max value of E-Field (V/m) induced in the brain by a TMS coil positioned (from left to right): 0.3 cm, 1.3 cm, 2.3 cm and 3.3 cm above the scalp. Right Panel: TMS coil with attached spacer on dummy head (coil ~ 3.3 cm from dummy head). (**C**) The positions of the motor hotspots over all 6 visits for each of 4 participants on their individual brains (P1—participant 1, P2—participant 2, etc.).

Here we sought to characterize how iTBS and cTBS modulate MEP amplitudes as compared to robust sham TBS (i.e., spacer-modified coil (Fig. 1B, Right Panel) held flat to the scalp in the same location as active TBS and with inactive masking) both across subjects, and within individuals across repeated neuronavigated TBS application. We hypothesized that iTBS and cTBS would increase and decrease corticospinal excitability, respectively, whereas sham TBS would not modulate corticospinal excitability. We expected these effects to hold at the group level across repeated visits and that individual responses to the protocols would also be reliable. In addition, in an effort to disentangle the impact of real and sham (including mechanical and electrical activation of peripherical nervous system) TMS effects, we corrected for the non-specific effects of cortical excitability by subtracting the modulation obtained by sham TBS at each time point from the active TBS data, thus better approximating the net effects of cTBS and iTBS on corticospinal excitability.

## Methods

### Participants

24 healthy participants (16 M; 18–49 years old; mean ± SD, 29.67 ± 10.60; all right-handed as assessed by a modified Edinburgh handedness questionnaire^22^) were enrolled in this study at the Berenson-Allen Center for Noninvasive Brain Stimulation at Beth Israel Deaconess Medical Center in Boston, MA. Participants reported no history of psychiatric or neurological diseases or chronic medical conditions and none were taking medications known to affect cortical excitability. All participants first provided written informed consent in accordance with the Declaration of Helsinki and all study procedures were approved by the Institutional Review Board at Beth Israel Deaconess Medical Center.

### Experimental procedures

Participants came to the lab for two iTBS, two cTBS, and two sham TBS visits for a total of six counter-balanced visits. Initial visits for each protocol were spaced at least 4 days apart, and repeat visits for a protocol were spaced at least a month apart. Visit order was kept consistent within a participant. So, if a participant’s first three visits in order were: cTBS, sham TBS, and iTBS, then the set of repeat visits was done in the same order. Participants completed the six visits on average over 6 weeks (range 4.3–8.3 weeks). All attempts were made to schedule all visits for a participant at the same time of day; however, if that was not possible due to scheduling constraints, then repeat visits of the same TBS protocols were ensured to be scheduled at the same time of day. All participants underwent individual high-resolution T1 MRI scans which were imported into the Brainsight™ TMS Frameless Navigation system (Rogue Research Inc., Montreal, Canada), and co-registered to digitized anatomical landmarks for online monitoring of coil positioning. Throughout the session, participants were comfortably seated in an adjustable chair. TMS was delivered with a Cool B65 figure-of-eight coil connected to a MagPro X100 stimulator (MagVenture A/S, Farum, Denmark). Monophasic TMS pulses were delivered with the coil oriented to induce a posterior–anterior current in the cortex. Motor hotspot was determined for each participant at each session, registered via neuronavigation (Fig. 1C), and used for all subsequent TMS. The hotspot was defined as the region where single-pulse TMS elicited the largest and most consistent MEPs in the first dorsal interosseous (FDI) muscle, with minimal simultaneous coactivation of the abductor pollicis brevis (APB) muscle^23^. Resting motor threshold was defined for the right first dorsal interosseous (FDI) muscle as the lowest stimulator intensity (percentage of maximum stimulator output; %MSO) that elicited MEPs ≥ 50 µV peak-to-peak amplitude at least 5/10 consecutive times. Active motor threshold was determined as the lowest %MSO that elicited MEPs of ≥ 200 µV at least 5/10 times while the participant minimally contracted the FDI muscle. Participants wore earplugs throughout any stimulation protocol to protect their hearing^24^. Baseline corticomotor excitability was evaluated pre-TBS with 120 single TMS pulses. The TBS protocol for that session was then applied, followed by six blocks of 60 single pulses at 5, 10, 20, 30, 50, and 60 min after the TBS (Fig. 1A). The interstimulus interval was jittered between 3 and 5 s within blocks of single pulses.

### MEPs

Motor evoked potentials (MEPs) were recorded from the FDI and APB muscles of the right hand. Ag–AgCl surface electrode-pairs were placed on the belly and tendon of the muscles, and the ground on the right ulnar styloid process. EMG data were amplified and digitized using a Powerlab 4/25 T data acquisition system (ADInstruments) at a sampling rate of 4000 Hz (bandpass filtered at 10–2000 Hz). EMG signals were continuously streamed by using LabChart software (LabChart 8.0) to monitor MEPs and epochs were recorded with a 150 ms window length covering from 50 ms before to 100 ms after the TMS pulse.

### TBS procedures

TBS was applied to motor hotspot, delivered as three pulse bursts at 50 Hz with 200 ms between bursts at 80% of AMT. iTBS consists of 2 s long trains of TBS with 8 s between each train for a total of 600 pulses over 190 s. cTBS delivers the TBS continuously over 40 s for a total of 600 pulses^3^. Participants were randomly assigned to receive either sham cTBS or sham iTBS across both sham visits. Sham cTBS and iTBS were administered on the scalp from the placebo side of the Cool-B65 A/P coil with a 3D printed 3.3 cm spacer additionally attached to the placebo side (MagVenture A/S, Farum, Denmark). The spacer was introduced since previous biophysical modelling work has demonstrated that a small percentage of the current (~ 5%) could still reach the cortex from the sham side of the coil^25^. The thickness of the spacer (3.3 cm) was chosen to reduce the current to negligible amounts (refer to Fig. 1B for a visualization of further modelling work and a mock set up of the sham TBS). Additionally, surface electrodes (Ambu Neuroline 715, Ambu A/S Baltorpbakken 13, DK-2750 Ballerup) placed approximately 1 cm below the inion delivered positive triangle current pulses (2.2 ms long in pulse width), proportional to the intensity (2-3 mA) of and synchronous with the TBS. The electrical stimulation was active across all TBS procedures and was done with the intention of blinding participants as to what kind of TBS they were receiving when they did not feel the ‘pulses’ from sham TBS.

### MRI data acquisition

A T1-weighted anatomical MRI scan was obtained in all participants and used for neuronavigation. Scans were completed on a 3T scanner (GE Healthcare, Ltd., United Kingdom) using a 3D spoiled gradient echo sequence: 166 axial-oriented slices for whole-brain coverage; 240-mm isotropic field-of-view; 0.937-mm × 0.937-mm × 1-mm native resolution; flip angle = 15°; TE/TR ≥ 2.9/6.9 ms; duration ≥ 432 s.

### Data analysis

Data were preprocessed using Microsoft Excel 2016. MATLAB *version 9.8.0* (R2020a) and the Statistics and Machine Learning Toolbox *version 11.7* (R2020a) (The MathWorks, Inc., Natick, MA, United States) were used for inferential statistical calculations. Peak-to-peak MEP values were taken as the difference between the maximum and minimum value from 20 to 50 ms after a single pulse within any one trial. Within any TMS block, any individual amplitudes greater than 2.5 standard deviations (SDs) from the mean were considered outliers and removed from that block. Mean MEP amplitudes for each post-TBS time point were expressed as the percentage change from baseline MEP amplitudes. If a participant’s percentage change from baseline for a particular post-plasticity block was 2.5 SDs from the group average for that time point, that participant’s data at that time point was removed as an outlier. In cases where participants were missing data at a time point, group mean data imputation was used. In total, 102/864 (11.8%) post-TBS time points were imputed: 34/864 (3.9%) time points differed more than 2.5 SD from the mean, 29/864 (3.4%) time points were missing due to time constraints, 14/864 (1.6%) time points were lost due to device malfunction, 13/864 (1.5%) time points were not useable either because of too few baseline MEPs or too few MEPs at the time point, and 12/864 (1.4%) time points were lost due to missing files.

A number of control analyses were performed including paired *t*-tests to determine whether (1) grand-averaged AMT changed from V1 visits to V2 visits, (2) raw baseline MEP values changed from V1 visits to V2 visits, or whether (3) coil displacement errors, as measured in mm from target, differed pre- or post-TBS within V1 visits and V2 visits. As well, all percentage change from baseline data was organized by session, i.e., by actual visit number (1–6), and a one-way ANOVA was performed to determine whether there was a session order effect regardless of TBS protocol. Then three one-way ANOVAs, one for each TBS protocol, were run to determine the effect of TBS protocol and session. Multiple comparisons between sessions within a TBS protocol were Bonferroni-corrected.

Participant’s percentage change from baseline data for each TBS protocol and each post-plasticity time point were entered into a repeated-measures ANOVA to test for the main effect of time (i.e., time points) and the interaction effect of TBS protocol (e.g., cTBS) by time. Multiple comparisons between time points within a TBS protocol were Bonferroni-corrected. Grand averages for each participant across time points were calculated for each TBS protocol by visit and Pearson correlation coefficients were calculated between visits. Then interclass correlation coefficients (ICCs) were calculated to determine the reliability with which each TBS protocol elicited its effects across repeat visits at each time point. ICCs were calculated using a two-way mixed-effects model, with fixed column (c) effects and random row (r) effects^26^:

$$\mathrm{ICC}\left(\mathrm{A},1\right)=\frac{\mathrm{MSr}-\mathrm{MSe}}{MSr+\left(k-1\right)* MSe+\frac{k}{n}*(MSc-MSe)}$$

where *ICC(A,1)* represents the degree of absolute agreement of measurements made under the two fixed levels of the column factor. *k* is the number of measurements per subject; n is the number of subjects; *MSr* is the mean square for rows (representing the individual subjects); *MSc* is the mean square for columns (representing the two visits); *MSe* is the mean square error (representing the residual sources of variance). ICC values < 0.25 are generally considered to indicate *very low to no reliability*, 0.25 ≤ ICC < 0.50 indicate *low reliability*, 0.50 ≤ ICC < 0.75 indicate *moderate reliability*, and ICC values ≥ 0.75 indicate *high reliability*^27,28^.

Each participant’s iTBS- and cTBS-modulated MEPs were corrected for sham effect by subtracting sham TBS percentage change from baseline from iTBS and cTBS percentage change from baseline for each corresponding time point. The sham-corrected data were entered into a repeated-measures ANOVA to test for the main effect of time and a TBS protocol by time interaction effect. Multiple comparisons between time points within a sham-corrected TBS protocol were Bonferroni-corrected. Pearson correlation coefficients were again calculated with grand averages of time points within a TBS protocol to measure the reliability of cTBS or iTBS at the participant level between visits with the effect of sham removed. All data were tested for normality with Kolmogorov–Smirnov tests. If any data were found to be non-normal, they were log-transformed before parametric testing.

## Results

### TBS protocol effects

**Figure 2:**
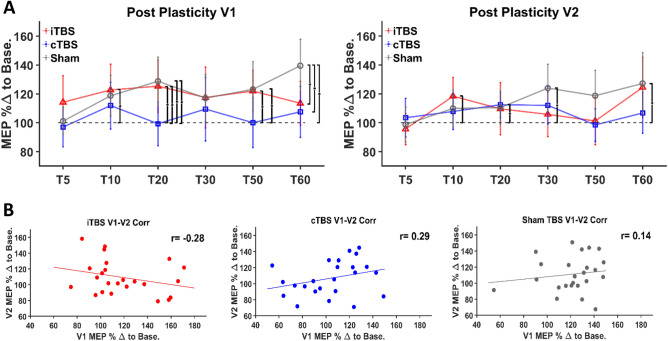
Group level and individual results. (**A**) Left and Right Panel: V1 and V2 of all TBS protocols respectively. Group-averaged MEP percentage change from baseline at each time point for each TBS protocol. Error bars denote ×2 standard error of the mean. Brackets indicate a significant difference (i.e. *p* < 0.05) between TBS protocols or a significant difference between a protocol and baseline (i.e. 100%, indicated by grey dotted line). (**B**) Left, Middle and Right Panel: Individual level scatter plots for each TBS protocol between repeat visits (from left: iTBS, cTBS, and sham respectively). On the x-axis is the participant’s response at V1 for a particular TBS protocol averaged across all time points and on the y-axis their response at V2 of that same protocol averaged across all time points. Fit lines denote least squares. *Abbreviations:* MEP %Δ to Base—Motor evoked potential percentage change to baseline.

The percentage change from baseline for each post-baseline time point within each protocol was entered into two repeated-measures ANOVAs (one for each visit) with TBS protocol as a predictor. There was a significant main effect of time in V1 (Fig. 2A, Left Panel) (*F*_5,345_ = 3.07, *p* = 0.010), and V2 (Fig. 2A, Right Panel) (*F*_5,345_ = 4.81, *p* = 3.6 × 10^–4^). There was a significant interaction effect between time and TBS protocol in V1 (*F*_10,345_ = 2.07, *p* = 0.027) but not in V2 (*F*_10,345_ = 1.84, *p* = 0.052). Bonferroni corrected paired *t*-tests confirmed that MEPs following iTBS were significantly larger than cTBS at T20 (*p* = 0.015) (Fig. 2a, Left Panel) and MEPs following sham were significantly larger than cTBS at T20 (*p* = 0.028), and T60 (*p* = 0.014). Additionally, modulation of MEPs was significantly larger following sham TBS than iTBS at T60 (*p* = 0.030). No significant differences were revealed between any of the time points between TBS protocols for V2.

The main effect of a TBS protocol was examined at each time point for both visits as compared to baseline (i.e. 100%, no change from pre-TBS pulses). Bonferroni-corrected two-tailed, one-sample *t*-tests with baseline corrected MEP responses at each time point compared to 100% revealed that MEPs following V1 sham TBS were significantly larger than baseline at T20 (*p* = 0.004), T50 (*p* = 0.021), and T60 (*p* = 1.61 × 10^–5^). MEPs following V1 iTBS were significantly larger than baseline MEPs at T10 (*p* = 0.033), T20 (*p* = 0.047), and T50 (*p* = 0.002). There were significant differences from baseline in V2 at T10 (*p* = 0.014) for iTBS, T20 (*p* = 0.036) for cTBS, and T30 (*p* = 0.048) and T60 (*p* = 0.047) for sham TBS.

### Intra-individual reliability of TBS protocols between visits

Correlations and interclass correlation coefficients (ICCs) were calculated to understand the reliability of TBS effects between visits. For each participant, within each visit and TBS protocol, percentage change from baseline data were averaged across all post-baseline time points. Each participant’s grand-averaged data from a V1 TBS protocol were correlated with the same TBS protocol from V2. There were no significant correlations within any of the TBS protocols between their respective visits iTBS: (r = -0.284, *p* = 0.179), cTBS: (r = 0.298, *p* = 0.158), and sham TBS: (r = 0.149, *p* = 0.304) (Fig. 2B). ICCs were calculated as a measure of the reproducibility of evoked responses between V1 and V2 for each TBS protocol at each time point. Percentage change from baseline data averaged within a time point for each participant was correlated at each time point between visits within a TBS protocol. After Bonferroni correction, the ICC for cTBS at T10 was moderately reliable (ICC: 0.51, *p* = 0.031) (Table 1) whereas there were no other significant ICCs for any of the other TBS protocols at any other time point.

**Table 1:** MEP percentage change from baseline (mean + SD) and reliability of TBS protocols.

Stim	TP	V1%Δ	V2%Δ	V1–V2	ICC	*p*-value
**iTBS**	T5	114.2 ± 39.2	95.6 ± 21.9	18.6 ± 47.1	− 0.09	1
	T10	122.7 ± 36.3	118.4 ± 26.4	4.3 ± 47.5	− 0.12	1
	T20	125.3 ± 42.5	109.6 ± 38.5	15.7 ± 63.6	− 0.22	1
	T30	117.5 ± 49.3	105.8 ± 31.4	11.7 ± 60.0	− 0.05	1
	T50	122.0 ± 25.9	101.2 ± 33.4	20.8 ± 46.1	− 0.16	1
	T60	113.5 ± 26.0	124.4 ± 42.3	− 11.0 ± 52.2	− 0.11	1
**cTBS**	T5	97.0 ± 32.0	103.4 ± 32.7	− 6.4 ± 39.9	0.24	0.742
	T10	111.9 ± 39.8	107.7 ± 30.6	4.2 ± 35.5	**0.51**	**0.031**
	T20	99.3 ± 33.8	112.4 ± 20.1	− 13.2 ± 36.2	0.14	1
	T30	109.4 ± 53.9	112.0 ± 31.5	− 2.6 ± 62.6	− 0.004	1
	T50	100.1 ± 31.5	98.4 ± 24.2	1.7 ± 43.9	− 0.23	1
	T60	107.6 ± 26.7	106.7 ± 34.2	0.82 ± 36.4	0.31	0.446
**Sham TBS**	T5	101.1 ± 19.8	98.7 ± 27.1	2.4 ± 30.8	0.16	1
	T10	118.7 ± 31.8	109.9 ± 31.9	8.8 ± 40.8	0.18	1
	T20	128.8 ± 35.7	110.5 ± 26.9	18.3 ± 48	− 0.14	1
	T30	117.0 ± 34.6	124.0 ± 40.4	− 6.9 ± 56.3	− 0.12	1
	T50	123.1 ± 34.9	118.7 ± 35.6	4.5 ± 38.3	0.42	0.122
	T60	139.5 ± 31.4	127.2 ± 45.6	12.4 ± 53.6	0.06	1

Bonferroni-corrected *p-values* < 0.05 and associated ICCs are bolded.

*Stim* TBS protocol, *TP* time point, *V1/2%Δ* visit 1/2 MEP percentage change from baseline, *V1–V2* difference of visit 1 and 2 MEP percentage change from baseline, *ICC* intraclass correlation coefficient.

### Control analyses

**Figure 3:**
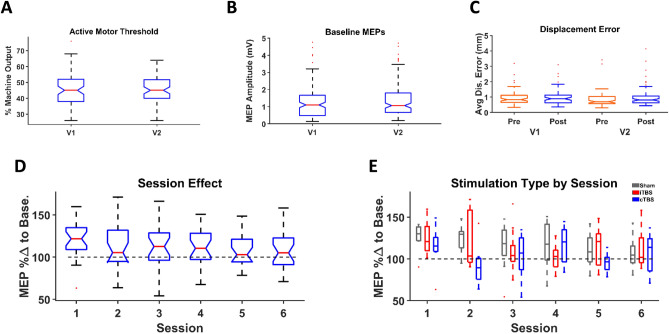
Control analyses. (**A**) Grand average of active motor threshold as a percentage of maximum machine output by V1 and V2 visits. (**B**) Grand average baseline MEP amplitudes (mV) by V1 and V2 visits. (**C**) Grand average of coil displacement error (mm) by V1 and V2 visits and separated into pre- and post-plasticity data. (**D**) Boxplot of post-TBS grand-averaged MEP percentage change from baseline by absolute visit number for all participants. (**E**) Box plot of post-TBS grand-averaged MEP percentage change from baseline by absolute visit number grouped by TBS protocols. *Abbreviations:* Avg Dis. Error - average displacement error. MEP %Δ to Base—motor evoked potential percentage change to baseline.

In order to elucidate the potential impact of confounding factors on TBS effects as well as TBS reliability, control analyses were carried out assessing variables such as baseline corticospinal excitability, TMS coil displacement error and practice/session effects. There was no significant difference in active motor threshold values, expressed as percentage of maximum stimulator output between V1 visits and V2 visits (Fig. 3A) (V1 visits: 45.58% ± 9.81% (*sd*) and V2 visits: 45.80% ± 8.89% (*sd*); *t*_70_ = − 0.31, *p* = 0.755). There was no significant difference between baseline MEP values for all V1 and V2 visits (Fig. 3B) (V1 visits: 1.31 mV ± 1.13 (*sd*) and V2 visits: 1.34 mV ± 1.01 (*sd*); *t*_141_ = − 1.04, *p* = 0.304). There were no significant differences between V1 visits pre- and post- and V2 visits pre- and post- average displacement errors (Fig. 3C) (V1 visits pre: 1.28 mm ± 1.83, V1 visits post: 0.99 mm ± 0.51, V2 visits pre: 1.04 mm ± 1.08, V2 visits post: 1.01 mm ± 0.65; *t*_68_ = 0.21, *p* = 0.837 and *t*_70_ = -1.06, *p* = 0.291 respectively). A repeated-measures ANOVA revealed no order effect of session (Fig. 3D) (*F*_5,115_ = 0.95, *p* = 0.45) and paired *t*-tests revealed no significant differences between any sessions after familywise corrections (15 comparisons, all *p'*s > 0.05). Three one-way ANOVAs revealed no order effect of TBS protocol and session (Fig. 3E) (iTBS: *F*_5,42_ = 0.52, *p* = 0.7615, cTBS: *F*_5,42_ = 0.77, *p* = 0.5768, and sham: *F*_5,42_ = 0.96, *p* = 0.4545) and *t*-tests revealed no significant differences between any sessions within a TBS protocol after familywise corrections (15 comparisons per TBS protocol, all *p'*s > 0.05). Additional post-hoc analyses investigated potential session one TBS protocol (e.g. sham TBS) carryover effects on sessions two and three. No significant session one TBS protocol carryover effects on session two or three were discovered. Detailed explanations of these analyses are included in “Supplementary Materials” (i.e., Figs. S2 and S3).

### Sham-corrected TBS protocol effects

**Figure 4:**
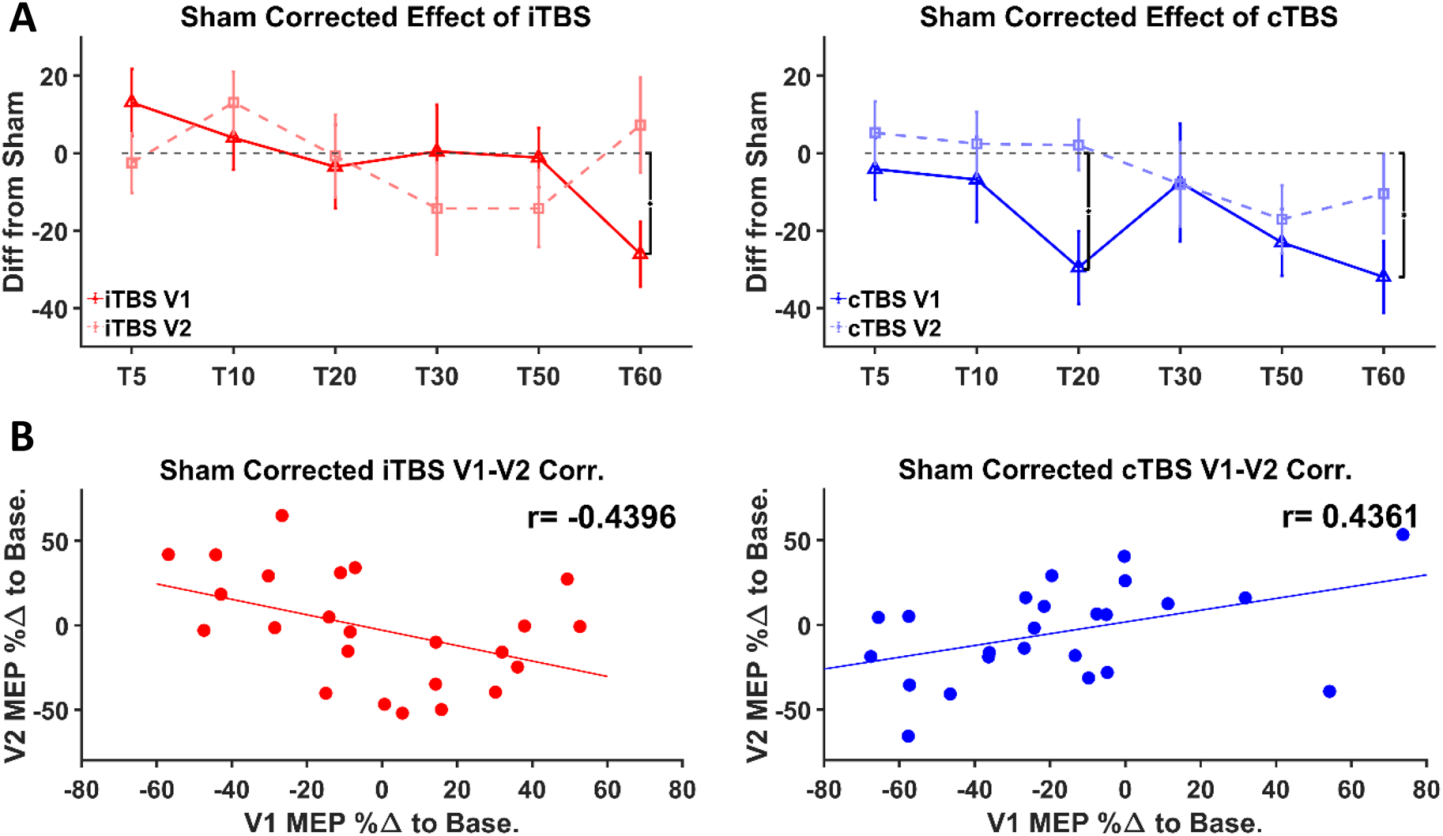
Sham-corrected effect of TBS. (**A**) Left and Right Panel: Group-averaged MEP percentage change from baseline at each time point for each visit with sham TBS subtracted out (from left to right: iTBS and cTBS, respectively). Error bars denote ×2 standard error of the mean. Brackets indicate a significant difference (i.e. *p* < 0.05) between a TBS protocol at V1 from baseline (i.e. 0, indicated by grey dotted line). (**B**) Left and Right Panel: Individual-level scatter plots for each TBS protocol between repeat visits with sham subtracted out (from left to right: iTBS and cTBS, respectively). On the x-axis is the participant’s response at V1 for that TBS protocol averaged across all time points and on the y-axis their response at V2 to that TBS protocol averaged across all time points with the equivalent sham responses subtracted out. Fit lines denote least squares. *Abbreviations:* Diff from Sham - Difference from sham, MEP %Δ to Base—motor evoked potential percentage change to baseline.

Given the lack of straightforward effects for TBS, the impact of potential placebo effects was further explored by subtracting the modulation of cortical excitability observed during sham TBS from iTBS and cTBS data. Specifically, percentage change from baseline data from each sham TBS post-baseline time point was subtracted by participant from iTBS and cTBS data at the same time points. These corrected data were then entered into a repeated-measure ANOVA with TBS protocol as a predictor. There was a significant main effect of time at V1 (*F*_5,230_ = 4.63, *p* = 4.75 × 10^–4^) but no significant interaction between TBS protocol and time (*F*_5,230_ = 0.51, *p* = 0.77). Bonferroni-corrected two-tailed, one-sample *t*-tests with sham-corrected MEP responses at each time point compared to 0 (i.e. baseline) revealed that MEP amplitudes following cTBS, were significantly smaller than baseline at T20 (*p* = 0.028) and T60 (*p* = 0.014) in V1. Following iTBS, MEP amplitudes were also significantly smaller than baseline at T60 (*p* = 0.03) in V1. There was a significant main effect of time at V2 (*F*_5,230_ = 2.38, *p* = 0.039) but no significant interaction effect between TBS protocol and time (*F*_5,230_ = 0.81, *p* = 0.55) (Fig. 4A). There were no significant differences from baseline for iTBS or cTBS at any of the time points in V2. The sham-corrected data were averaged for each participant across each time point within a TBS protocol for each visit and Pearson correlation coefficients were calculated for these grand averages between V1 and V2. Participant responses post-cTBS and -iTBS were significantly positively correlated (r = 0.4361, p = 0.033) and negatively correlated (r = − 0.4396, p = 0.032) respectively between visits (Fig. 4B).

### TBS protocol responders versus non-responders

**Figure 5:**
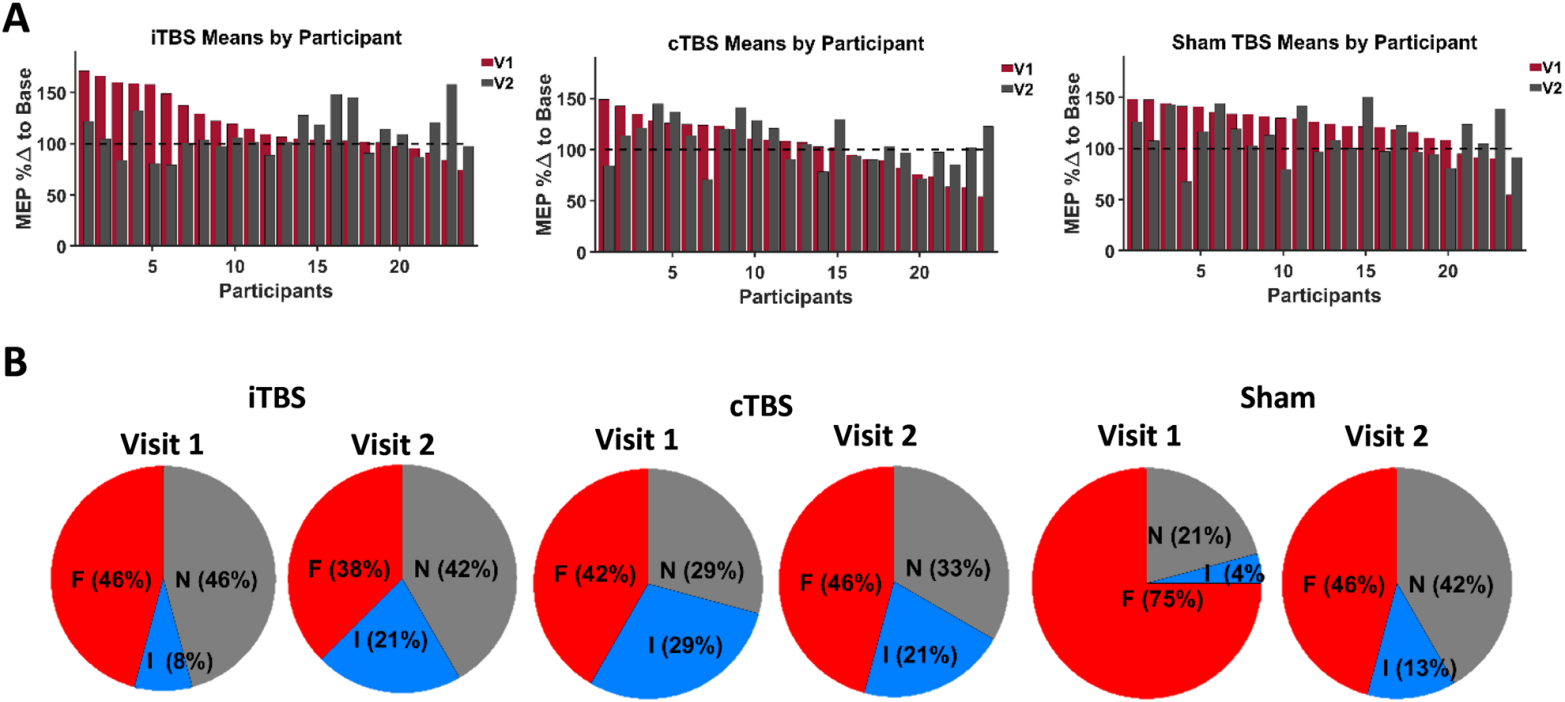
Individual responses and responder types. (**A**) Left, Middle, and Right Panel: Bar charts of individual participant (x-axis) mean MEP percentage change from baseline across all time points (y-axis) in V1 (maroon) and V2 (dark grey) of each TBS protocol (from left: iTBS, cTBS, and sham respectively). Participants are organized in descending order according to their V1 responses. *Abbreviations:* MEP %Δ to Base—motor evoked potential percentage change to baseline. (**B**) Left, Middle, and Right Panel: Pie charts of percentage of facilitators (F (red), ≥ 110%), inhibitors (I (blue), ≤ 90%) and non-responders (N (grey), 110% < µ > 90%) grouped by TBS protocol and visit number (from left: iTBS, cTBS, and sham respectively).

Participants in TMS studies can be classified as facilitators, inhibitors, or non-responders if they have a MEP percentage change from baseline (µ) µ ≥ 110%, µ ≤ 90% or 110% < µ > 90%, respectively (Fig. 5B)^19^. Furthermore, participants can be classified as responders if MEPs are facilitated after iTBS or if they are inhibited after cTBS, as these are the expected effects of these TBS protocols. These classifications are typically used after only one TMS visit but are rarely explored in studies with repeat sessions^1^. As well, it is generally assumed that while there may be between-participant variability in response to a particular TBS protocol, a participant will respond similarly across visits of the same TBS protocol^15^. To test these assumptions, the consistency of categorizations between repeat visits was examined closely.

Given two study visits and three possible categorizations, it is expected by chance that ~ 11% of participants will be labelled as responders across either both iTBS or both cTBS visits. Only 2/24 (~ 8.3%) participants were categorized as responders across both visits of iTBS and only 2/24 (~ 8.3%) participants were categorized as responders across both visits of cTBS. Therefore, it is no better than chance that a participant will be labelled as a responder across both visits of either cTBS or iTBS protocols. However, a participant may experience a reliable non-canonical modulation in MEPs after TBS.

Only iTBS and cTBS are hypothesized to have a modulatory effect, as compared to sham TBS, and only those labelled as a facilitator or inhibitor in V1 are further examined as non-response is by definition not a modulation of MEPs. By chance, given these further restrictions, it is expected that 50% of respondents would remain in the same category between visits. 2/13 (~ 15%) participants were categorized reliably as facilitators or inhibitors across iTBS visits, less than would be expected by chance. 10/17 (~ 59%) were categorized reliably as facilitators or inhibitors across both cTBS visits, as expected by chance, binomial *p* (one-tailed) = 0.149. Again, it is no better than chance that a participant will be reliably categorized between visits of the same TBS protocol for either iTBS or cTBS protocols. Interestingly, facilitation was more frequently reliable after cTBS study visits (8/17) than iTBS study visits, (2/13). Additionally, facilitation was the more frequently reliable response post-cTBS than inhibition (2/17), the canonical response.

Two chi-square analyses were run to determine whether there was a difference between TBS protocols in terms of how participants were categorized at each visit (Fig. 5B). Classifications as either a facilitator, non-responder, or inhibitor were entered into two 3 × 3 tables (one table per visit, rows were categorization, columns were TBS protocol). The chi-squared analysis revealed that in V1 there was a significant difference in how participants were categorized dependent on the TBS protocol (*X*^*2*^_4,24_ = 11.56, *p* = 0.009) but by V2 there was no significant difference (*X*^*2*^_4,24_ = 1.16, *p* = 0.162).

## Discussion

TBS has been shown to modulate cortical excitability for 5–10 min after a single application^12^. While a few studies have demonstrated longer lasting MEP modulation across repeated iTBS visits^2,16^ or reliable early effects on MEPs (e.g., 5 min after TBS) in the case of cTBS^15^, other studies exhibit weak to null iTBS modulation overall^18^ or no consistent iTBS modulation between visits^13,14,17^. Given the lack of consistent results for TBS protocols, we sought to clarify the reliability of TBS protocols overall by administering iTBS, cTBS and sham TBS within the same cohort of healthy volunteers across six counterbalanced visits. In this study, cortical excitability was increased after iTBS and sham TBS in V1, whereas cTBS had no effect as compared to baseline MEPs. None of the TBS protocols reliably modulated MEPs across study visits. The effects of TBS protocols, the relevance of sham stimulation in TBS experiments, and the potential implications of our findings for reliability and efficacy of TBS protocols are discussed below.

In V1 iTBS significantly increased MEPs as compared to baseline at T10, T20, and T50, consistent with the literature^2,12,13^. Additionally, iTBS increased the size of MEPs at T10 in V2, which replicates a finding of early facilitation at a second iTBS visit^2^. Given published studies, cTBS was predicted to decrease the size of MEPs post-TBS^3,15^, however there were no significant changes as compared to baseline at any time points in V1 and there was a significant facilitation of MEPs at T20 in V2. The null effects of cTBS at V1 mirrored results observed in Hamada et al.^1^. Additionally, the facilitation of MEPs observed in V2 at T20, has a similar corollary in Vernet et al.^15^ where a near significant facilitation of MEPs post-cTBS was observed in visit one at T30 of that study. Contrary to expectations and previous null effects of sham TBS on MEPs^13,14,19^, post-sham TBS MEPs were larger than baseline MEPs at T20, T50 and T60. Post-sham TBS MEPs were even larger than post-iTBS MEPs at T60 in V1. MEPs were similarly facilitated post-sham TBS in V2 at T30 and T60. This noticeable difference between this study and others could speculatively be accounted for by a strong placebo effect given that our study used a robust sham including that the spacer-modified coil was placed flat at the site of active stimulation and it utilized inactive masking, features that were individually absent in previous studies of TBS-evoked MEPs where sham TBS was used^13,14,19^. Most aftereffects of TBS protocols are seen in the first visit and these effects are generally weaker or are not present in follow-up visits^13,28^.

When post-sham MEPs were subtracted at each time point from post-cTBS and -iTBS MEPs—as a proxy of unspecific effects of repeated single TMS pulses and/or placebo—the results were inverted. With the effect of sham removed, a significant reduction in amplitude of MEPs as compared to baseline was observed after cTBS at T20 and T60 in V1 (as is the predicted effect of cTBS). However, there were no significant changes from baseline in MEPs after cTBS in V2. With the effect of sham removed, iTBS demonstrated an inhibitory effect on MEPs at T60 in V1 but no other significant modulations at any other time point in either visit. Therefore, with the effect of sham removed, iTBS no longer had its predicted effect of facilitating MEPs in V1, yet cTBS had its predicted effect of inhibiting MEPs. This might suggest an underlying effect of increased corticospinal excitability over time^29^ or that receiving successive single pulses over the duration of the visit increases corticospinal excitability^30^. This interpretation of the results suggests that cTBS had its canonical effect in the first visit, whereas iTBS had no modulatory effect on MEPs over and above time/successive single pulses of TMS. Regardless, such a large facilitation after sham TBS in the first and second visit was intriguing and unexpected. As such, variable sham aftereffects could be indicative of poorly understood underlying mechanisms^30^ that mask the true effects of verum TBS. These results and their interpretation are exploratory and require further experimentation with sham TBS to fully understand their implications.

Post-TBS MEP grand averages (across time points) within a participant and TBS protocol were correlated across visits. No protocol demonstrated any significant correlation between V1 and V2, mirroring the plurality of reported data on the lack of intra-individual reliability of TBS protocols across visits^13–15,18,28^. However, sham-corrected cTBS and iTBS grand-averaged MEPs were, respectively, significantly positively and negatively correlated between visits. Such results point to regression to the mean or metaplastic effects related to TBS exposure.

Interclass correlation coefficients were calculated to measure the reliability of TBS protocols at individual time points between repeat visits. Post-cTBS MEPs were significantly correlated between V1 and V2 at T10 with an ICC value of 0.51, indicating moderate reliability. This result is consistent with other studies which find moderate post-cTBS MEP reliability at early time points (e.g., T5 or T10^15,28^). Despite the reliability, the MEPs post-cTBS at T10 were un-modulated compared to baseline MEPs. In addition, there were no other significant ICCs between any other time points for any TBS protocols between the two visits.

Overall, in this study, cTBS and iTBS did not reliably evoke their predicted effects between visits. Such results could be due to a regression to the mean^31^. Indeed, with the effect of sham removed, a significant negative and positive correlation between visits for iTBS and cTBS is observed, respectively. Also, as is evident in Fig. 5A, especially for iTBS, if post-TBS MEPs were facilitated for a participant at V1, they tended to be inhibited at V2 and vice versa. Regression to the mean could also be due to metaplastic effects^1^. Initial exposure to TBS could induce hypothesized effects in a portion of the participant population as observed in this study and others^1^. However, the TBS is temporally uncorrelated with any external or endogenous signal, so its effect on brain activity may wane through continued exposure. Additionally, while it could be argued that there is some minimal replicability between visits at least at early time points for cTBS^15^ and iTBS, several minutes of uncorrelated modulation is unlikely to induce lasting anatomical or functional changes. As these protocols, especially iTBS^9,10^ are used therapeutically, the lack of consistency in effect and duration of modulation is of note, as therapeutic effects are derived theoretically from the reliable and sustained modulation of brain activity across multiple visits. However, there is evidence that a ‘noisy’ brain is a healthy one^32,33^, and some have argued that the therapeutic effect of non-invasive forms of brain stimulation may actually be derived from non-specific global modulation of neural activity^34^, i.e., ‘by increasing neural noise’. Therefore, further experimentation to understand the mechanism underlying clinical efficacy in treating disorders such as depression is needed.

Classification as either a facilitator, inhibitor or non-responder is significantly different between TBS protocols in V1; however, there is no significant difference in V2. As well, the canonical effects of iTBS and cTBS protocols occur most prominently in the first visit. By the second visit of any TBS protocol, participants separate almost equally into facilitator and non-responder categories for all TBS protocolss, while the remainder funnel into the inhibitor category (Fig. 5B). It was expected that facilitation would be the most common categorization after iTBS and inhibition would be the most common categorization after cTBS, especially in V1. As well, these canonical responses should be most frequent with the respective protocol as compared to the other protocols. However, this is not the case. While inhibition is most common in cTBS (29%) in the first visit, as compared to the other protocols, it is not the most common response for that protocol, which is facilitation (42%). While facilitation is the most common response after iTBS (46%), sham TBS induces even more participants to facilitate in visit one (75%) (Fig. 5B). These findings are supported by other studies that saw similar rates of expected and unexpected responses to TBS procedures^1,35^. Despite interindividual variability in iTBS and cTBS aftereffects, there could be robust intraindividual reliability. But this does not bear out as canonical or non-canonical categorizations after iTBS or cTBS were no more likely than chance to occur within a participant across repeat visits.

Our findings of facilitation of MEPs following sham TBS necessitates further discussion on the topic of placebo effects. Placebo effects can be defined as the positive therapeutic responses attributed to the context surrounding administration of an intervention (e.g., environmental cues and their associated expectations) rather than the intervention itself^36^. Though traditionally viewed as a nuisance, recent neuroimaging and neurophysiological studies have demonstrated that placebo effects can meaningfully modulate brain regions/networks and neurotransmitter systems^37^. In research trials with randomization to a placebo group, the overall placebo “response” includes placebo effects but also includes spontaneous improvement, regression to the mean, Hawthorne effects and other factors^36^. Like most studies, our design did not include a “no treatment” control group (receiving nothing) and thus it is not possible to delineate the relative contribution of the above factors to the sham TBS group response. It is also important to note that beyond a description of the mechanisms of TMS and TBS and safety concerns, subjects’ expectations of TMS or TBS were not explicitly modified. This is different from a conventional treatment trial that may directly promote positive expectation of improvement on a given metric; however, it is possible that subjects may have implicitly generated such an expectation. Furthermore, the elaborate and intensive technology associated with TMS procedures may elicit particularly large placebo responses^38^ (including a sham rTMS-induced placebo effect larger than the therapeutic benefit from the active rTMS group^39^) and these responses have been found to be increasing with time to present^40^. More sophisticated sham TMS devices (leading to improved blinding) may be one of many factors contributing to this trend.

Indeed, we contend that we observe such a strong faciliatory effect on MEPs post-sham TBS primarily due to placebo effects, as we have employed a robust sham procedure where the coil is held flat on the scalp at the same location as active TBS while using an inactive mask. We expect that these highlighted differences in sham TBS protocol could have led to a strong placebo effect not observed in other studies that employed sham TBS as a comparison to the effects of TBS protocols on MEPs^13,14,19^. However, this contention is speculative as we did not directly test or manipulate for placebo effects, and further experimentation with sham TBS would be necessitated to tease this out. Another contributing factor to the strong faciliatory effect observed after sham TBS could be the cumulative effects of single TMS pulses. Pellicciari et al. demonstrated that single-pulse TMS can induce cumulative increases in corticospinal excitability across multiple stimulation blocks within a session^30^. Julkunen et al. also demonstrated the increase of MEP amplitude as the number of stimuli increased, especially with shorter inter-trial intervals (ITIs) (i.e. 1–3 s and 3–5 s)^29^. Given that our single-pulse ITI was jittered from 3 to 5 s, common in TBS literature^1,35^, this further bolsters the contention that increases in corticospinal excitability after sham TBS could be attributed to single TMS pulses that increase the amplitude of MEPs over time. Speculatively, the cumulative effect of single pulses was observed after the sham protocol precisely as the protocol does not modulate cortical excitability and so the unadulterated effect of single pulses could be observed. However, cTBS and iTBS are intended to have some modulatory effect on brain activity and so the cumulative effect of single pulses could interact in such a way as to complement or interfere with the TBS-induced modulation of corticospinal excitability. Therefore, the cumulative effect of single pulses could be difficult to observe after cTBS and iTBS due to competing modulations. Ultimately, it is difficult to delineate the source(s) of the post-sham TBS faciliatory effect on MEPs within this study. So, we speculate it is due to the confluence of a strong sham effect and the cumulative effect of single TMS pulses.

A few limitations and caveats in our design must be noted and considered for future investigations. For instance, the TBS protocols were only tested at one repeat study visit, which limits extrapolations on the reliability of iTBS and cTBS over multiple repeat visits. Ideally, future studies will examine iTBS and/or cTBS over multiple repeat visits in a design that more closely models clinical application (i.e., multiple repeat visits over several weeks). Conceivably, individuals respond to TBS in a periodical fashion, generally reclassifying as responders at a better than chance rate. The observed facilitation of MEPs after sham TBS could hypothetically be attributed to cervical stimulation as direct current cervical stimulation has been shown to induce an increase in TMS-evoked MEPs after the period of stimulation^41^. However, these results are not consistent across studies, with a couple studies showing no effect of cervical stimulation with identical parameters^42,43^. Importantly, parameters used in this study differ considerably from the aforementioned studies as both the electrodes in this study were arranged posteriorly with one above the other, as opposed to in an anterior–posterior arrangement^41–43^. Also, the period of TBS pattern matched electrical stimulation was considerably shorter in this study, either lasting 1 min (sham cTBS) or 5 min (sham iTBS) versus 20 min of direct current stimulation^41–43^. So, if there was an effect of cervical stimulation then we may expect to see differences between sham cTBS and iTBS given the difference in duration of cervical stimulation, however no significant differences were observed between the two at any time point at either visit (“Supplementary Materials”, Fig. S1). Moreover, cervical stimulation was applied across all TBS protocols; so if there was an effect of cervical stimulation then it should have affected all protocols equally and we may expect to observe iTBS-facilitated MEPs above those of sham TBS, which was not the case.

MEPs themselves may be an imperfect measure for changes in cortical excitability as they are a downstream measure of ‘cortical’ excitability. Plausibly, the effects of TBS are more robust or reliable, however are difficult to capture due to how its effects are measured. TMS-evoked potentials (TEPs) are a promising measure of cortical excitability with a larger parameter space^11^ and may therefore prove to be a more direct and richer measure of TBS aftereffects than MEPs.

Importantly, we are able to rule out some explanations for our results as there were no differences in targeting, baseline MEP values, or AMT values between visits. All study visits were held at the same time of day for each participant, ruling out effects that vary throughout the day such as cortisol levels, however actual cortisol measurements could improve this aspect in future studies. Repeat study visits occurred about a month apart but visits of different types could occur within about a week time period (at least 3 days between visits), theoretically leaving enough time for carryover effects to be negated. Several analyses of session one carryover effect were not significant, bolstering the previous claim (Supplementary Materials, Figs. S2 and S3).

A future placebo-controlled study could incorporate a TBS-free condition in which there is a TBS-timed break in between baseline and ‘post-TBS’ pulses. Such a condition could delineate time-varying effects of single TMS pulses and serve as an additional comparison against chosen TBS protocols. Additionally, expectation effects could also be more thoroughly appreciated as the results from the sham and the TBS-free conditions should theoretically be identical.

Our data demonstrate considerable variability between and within participants in post-TBS measures, on top of an overall lack of test–retest reliability, questioning TBS use as a tool to induce reliable long-term plasticity in the human brain. Moreover, sham TBS also demonstrated an impact on corticospinal excitability over time, suggesting placebo effects should be accounted for in TBS protocols.

## Supplementary Information

Supplementary Figures.
