# Supplementary material for: Sham-derived effects and the minimal reliability of theta burst stimulation
Source: Sci Rep. 2021 Oct 27;11:21170. doi: 10.1038/s41598-021-98751-w (PMC8551312; doi:10.1038/s41598-021-98751-w)
Supplement: Supplementary file 1 — Supplementary Figures. [file 41598_2021_98751_MOESM1_ESM.docx]

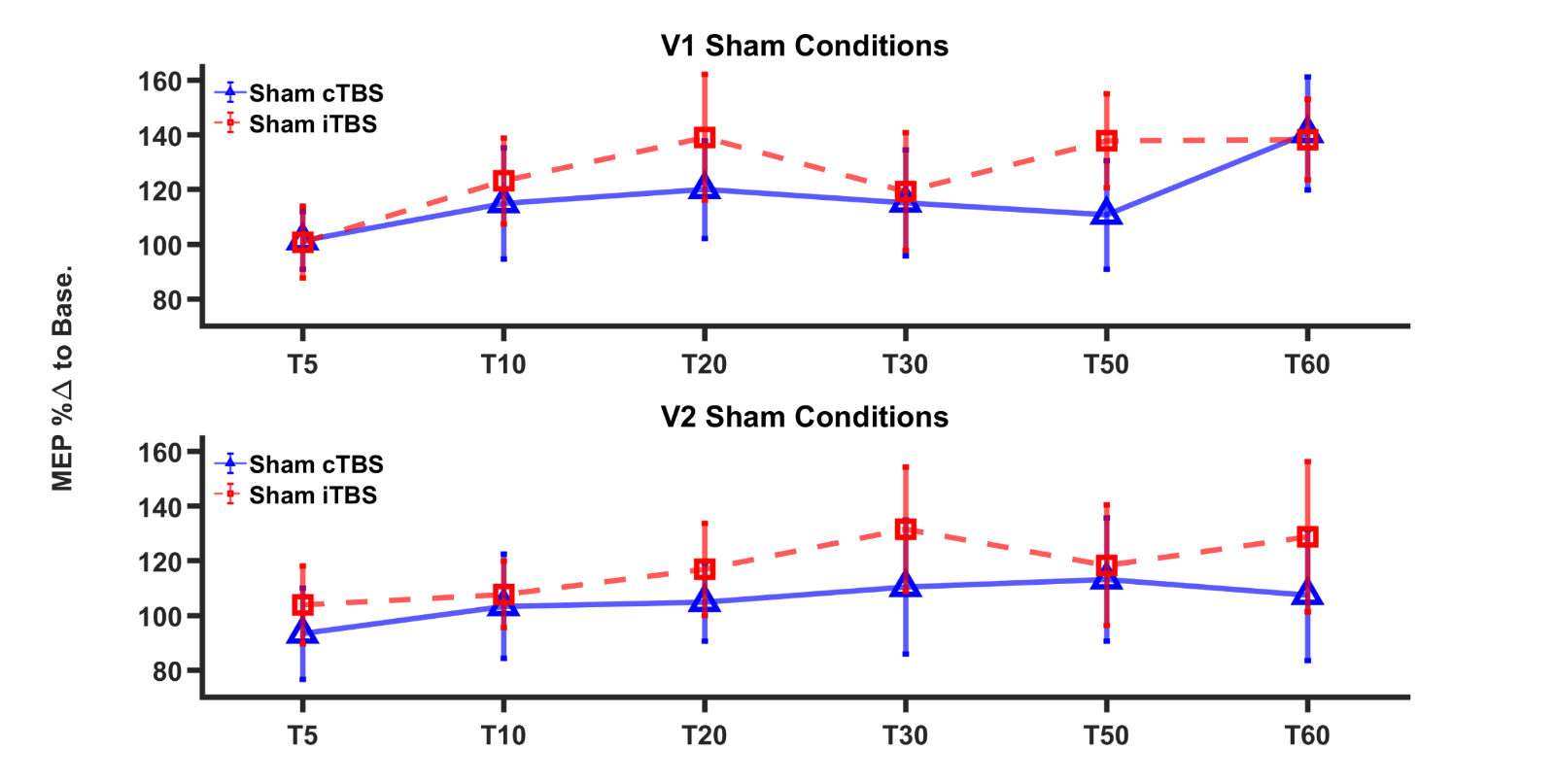
**Figure S1 – Sham Conditions** Group-averaged MEP percentage change from baseline at each time point for each sham condition (i.e. Sham cTBS & Sham iTBS) at visit one (V1) (top graph) and visit two (V2) (bottom graph) respectively. Error bars denote 2x SEM. *Abbreviations*: MEP %Δ to Base. – Motor evoked potential percentage change to baseline.

A repeated-measures ANOVA was conducted of post-sham cTBS and iTBS percentage change from baseline MEPs at V1 and V2 with time as the response variable and sham TBS condition as the predictor. There was a significant main effect of time (*F*_5,110_ = 3.47, *p*= 0.006), but no time by sham TBS condition interaction (*F*_5,110_ = 1.24, *p*= 0.29) at V1. There was no main effect of time (*F*_5,110_ = 0.67, *p*= 0.646) or interaction effect of time and sham TBS conditions at V2 (*F*_5,110_ = 0.35, *p*= 0.35). The main effect of time post-sham TBS at V1 is expected as it replicates the finding that MEPs tend to increase with time after sham TBS in V1. No significant differences were observed between baseline and any of the time points at either of the two visits, after Bonferroni corrections.


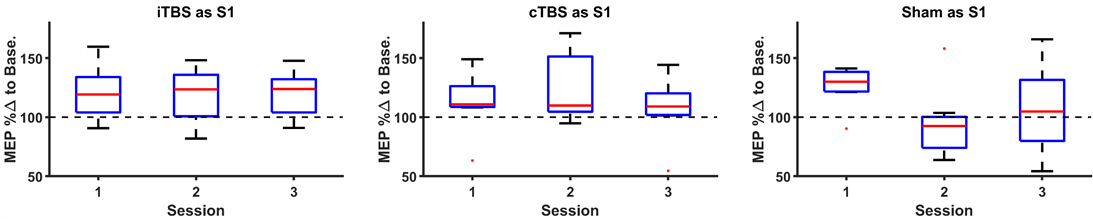


**Figure S2 – Effect of First Session TBS Protocol** Boxplots of grand-averaged post-TBS percentage change from baseline MEPs, grouped by participants’ first session TBS protocol compared to the participants’ following two sessions. The following two sessions include data regardless of what TBS protocol those participants experienced for each of those sessions. For example, if participants had iTBS in their first session, sessions two and three would have a mix of sham TBS and cTBS post-plasticity data (‘iTBS as S1’). Red dots indicate outliers (more than 1.5 times the interquartile range from the top or bottom of the box). *Abbreviations*: S1 – Session one, MEP %Δ to Base. – Motor evoked potential percentage change to baseline.

Participants were grouped based on their first session TBS protocol (e.g. iTBS) and three one-way ANOVAs were performed on post-TBS percentage change from baseline MEPs averaged across time points within each of their first three sessions to determine if the protocol on session one had an effect at session two or three. There was no effect of sham as session one (*F*_2,21_ = 2.6, *p*= 0.098), no effect of iTBS as session one (*F*_2,24_ = 0.03, *p*= 0.972), and no effect of cTBS as session one (*F*_2,18_ = 0.74, *p*= 0.491). After Bonferroni corrections (three comparisons per one-way ANOVA), none of the paired *t*-tests showed significant differences between any of the sessions as grouped by first session TBS protocol (e.g. sham as session one compared to session two (same participants): *t*_7_=2.99, *p*=0.061).


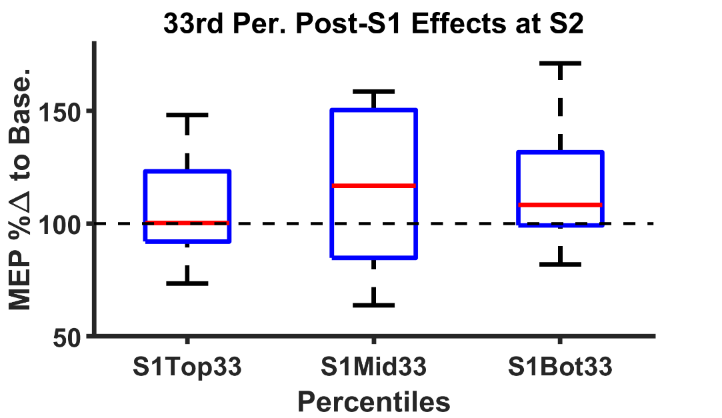

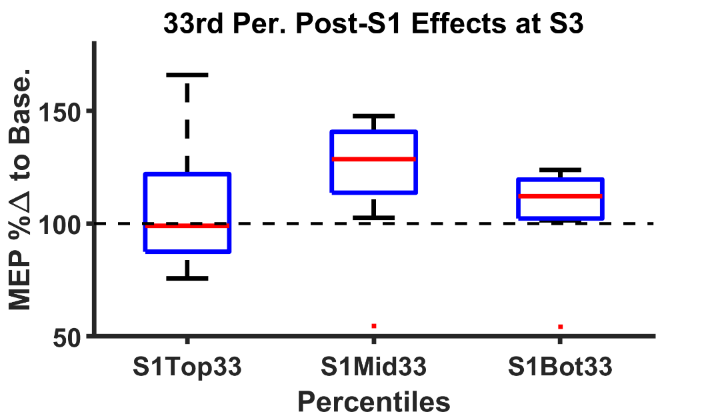

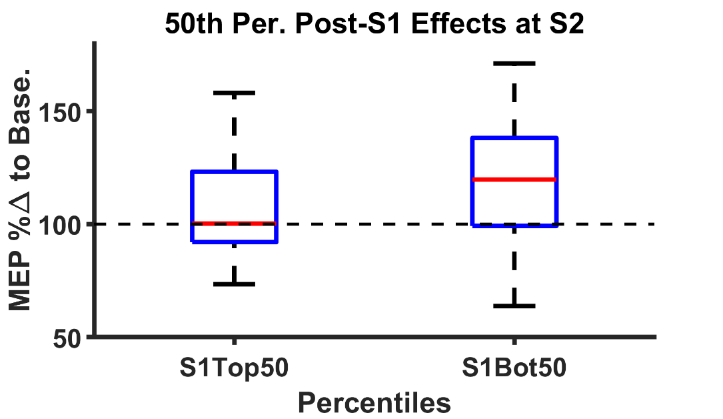

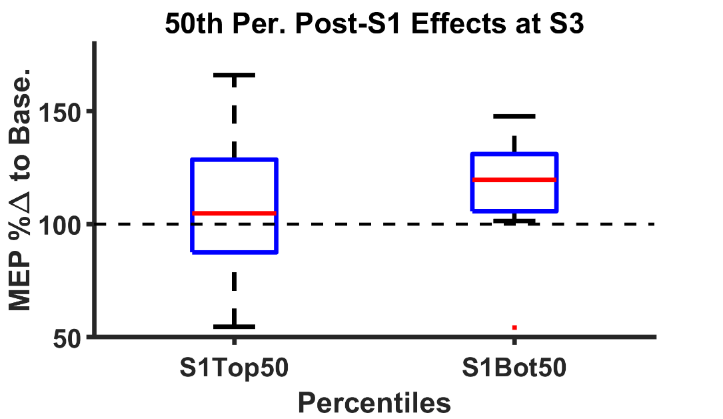


**A**

**B**

**Figure S3 – Effect of Post-TBS Response Percentile Grouping in First Session** Boxplots of grand-averaged post-TBS percentage change from baseline MEPs organized by percentile of TBS response in the first session. *A. Left & Right Panel* – 50^th^ percentile post-TBS MEP response in session one, at session two and three respectively. *B. Left & Right Panel* – 33^rd^ percentile post-TBS MEP response in session one, at session two and three respectively*.* Red dots indicate outliers (more than 1.5 times the interquartile range from the top or bottom of the box). *Abbreviations*: S1/2/3 – Session one/two/three, Per.- Percentile, S1Top50/33 – Top 50^th^ /33^rd^ percentile post-TBS MEP response in S1, S1Mid33- Middle 33^rd^ percentile post-TBS MEP response in S1, S1Bot50/33 – Bottom 50^th^/33^rd^ percentile post-TBS MEP response in S1, MEP %Δ to Base. – Motor evoked potential percentage change to baseline.

To determine if there was a strong/weak responder effect of the first session, two analyses were run. One analysis separated the participants into 50^th^ percentiles and the second divided them into 33^rd^ percentiles based on their post-TBS MEPs averaged across all time points at session one. Between group comparisons were done with post-TBS MEPs averaged across all time points at session two and three to determine if there were aftereffects of the first session. For the 50^th^ percentile analysis, two, two-sample *t*-tests were run and there were no significant differences between the top 50^th^ and bottom 50^th^ percentile session one groupings at either session two (*t*_22_=-1.0, *p*= 0.329) or session three (*t*_22_=-0.94, *p*= 0.355). For the 33^rd^ percentile analysis, two, one-way ANOVAs revealed no significant effect of session one percentile grouping at session two (*F*_2,21_ = 0.27, *p*= 0.766) or session three (*F*_2,21_ = 0.73, *p*= 0.492). *t*-tests revealed no significant differences between any of the three groups at either session two or three. Lack of significant results from the session one aftereffect analyses are reassuring as this could be interpreted as evidence that TBS protocol crossover effects between sessions were minimized or non-existent.
